# *In Vitro* Study of Endothelial Cell Morphology and Gene Expression in Response to Wall Shear Stress Induced by Arterial Stenosis

**DOI:** 10.3389/fbioe.2022.854109

**Published:** 2022-04-13

**Authors:** Lizhong Mu, Xiaolong Liu, Mengmeng Liu, Lili Long, Qingzhuo Chi, Ying He, Yue Pan, Changjin Ji, Ge Gao, Xiaona Li

**Affiliations:** ^1^ Key Laboratory of Ocean Energy Utilization and Energy Conservation of Ministry of Education, School of Energy and Power Engineering, Dalian University of Technology, Dalian, China; ^2^ Schood of Chemical Engineering, Dalian University of Technology, Dalian, China; ^3^ Ningbo Institute, Dalian University of Technology, Ningbo, China; ^4^ School of Biomedical Engineering, Capital Medical University, Beijing, China; ^5^ The First Affiliated Hospital of University of Science and Technology of China, Hefei, China; ^6^ Key Laboratory of Industrial Ecology and Environmental Engineering (MOE), School of Environmental Science and Technology, Dalian University of Technology, Dalian, China

**Keywords:** vascular stenosis, silicone-endothelial cell model, wall shear stress, inflammatory factor, EC morphology

## Abstract

**Objectives:** We examined the correlation between changes in hemodynamic characteristics induced by arterial stenosis and vascular endothelial cell (EC) morphology and gene expression in straight silicone arteries.

**Materials and methods:** Transparent silicone straight artery models with four degrees of stenosis (0, 30, 50, and 70%) were fabricated. Particle image velocimetry was performed to screen silicone vessel structures with good symmetry and to match the numerical simulations. After the inner surface of a symmetric model was populated with ECs, it was perfusion-cultured at a steady flow rate. A computational fluid dynamics (CFD) study was conducted under the same perfusion conditions as in the flow experiment. The high-WSS region was then identified by CFD simulation. EC morphology in the high-WSS regions was characterized by confocal microscopy. ECs were antibody-stained to analyze the expression of inflammatory factors, including matrix metalloproteinase (MMP)-9 and nuclear factor (NF)-*κ*B, which were then correlated with the CFD simulations.

**Results:** As the degree of vascular stenosis increases, more evident jet flow occurs, and the maximum WSS position moves away first and then back. ECs were irregularly shaped at vortex flow regions. The number of gaps between the cells in high-WSS regions increased. The MMP-9 and NF-*κ*B expression did not differ between vessels with 30 and 0% stenosis. When arterial stenosis was 70%, the MMP-9 and NF-*κ*B expression increased significantly, which correlated with the regions of substantially high WSS in the CFD simulations.

**Conclusion:** Stenotic arteries induce hemodynamic stress variations, which contribute to differences in EC morphology and gene expression. A high degree of vascular stenosis can directly increase inflammatory factor expression.

## Introduction

Atherosclerosis, one of the most common arterial diseases, seriously threatens human health. Artery stenosis caused by the presence of plaque is its typical feature (Gao et al., 2021), which often gives rise to the cardiovascular and cerebrovascular diseases, such as coronary atherosclerosis and carotid artery stenosis (Gorelick et al., 2008). An aneurysm is a local pathological congestive dilation of blood vessels caused by disease or weakening of the blood vessel wall. It is characterized by local arterial dilation and presents as a thin, distensible sac that is at risk of rupture (Sforza et al., 2009).

Post-stenotic dilation of artery is a common phenomenon in clinical diagnosis. Multiple stenoses are common in the arteries as are post-stenotic dilation of the artery (Moore, 1990). Post-stenotic dilatation of the coronary arteries can occur at high flow rates, possibly due to the wall shear stress following the constriction (Pincombe et al., 1999). In addition, clinical statistics show that in patients with intracranial artery stenosis, the proportion of the aneurysm with proximal stenosis is about 12.3%, significantly higher than the general incidence of 2–6% of intracranial aneurysm in the population (Rinkel et al., 1998). Clinical data show that *de novo* cerebral aneurysm formation is often accompanied by some degree of proximal artery stenosis (Jou et al., 2010; Kono et al., 2013; Ferns et al., 2011; Kono et al., 2014). Owing to jet flow caused by stenosis, the maximum WSS and WSSG at the aneurysm initiation site were approximately doubled and tripled, respectively (Kono et al., 2014). However, it is still unclear about the mechanobiological mechanism of how artery stenosis causes the dilation of the post-stenotic artery and how genetic expression variations respond to arterial stenosis.

It is known that the occurrence and growth of aneurysms are thought to be closely associated with abnormal hemodynamic changes, which regulate vascular biology and pathology. Interactions between biological and hemodynamic factors can cause complex arterial wall remodeling (Meng et al., 2007). Arterial endothelial cells (ECs) physiologically respond to wall shear stress (WSS) and WSS gradients (WSSGs). Computational fluid dynamics (CFD) is commonly used to evaluate hemodynamic flow and WSS/WSSG in the patient-specific vascular geometry under physiological conditions. Many studies have found that aneurysms predominantly occur at regions of high WSS (Kondo et al., 1997; Alfano et al., 2013) and that aneurysm initiation sites are correlated with high WSS (Chen et al., 2013), especially with high positive WSSGs (Metaxa et al., 2010). A major drawback of existing CFD studies is that the methods used have made it difficult to directly link the pathological effects of hemodynamic variables, such as WSS and WSSG, on biological tissues (Lu et al., 2011; Luo et al., 2011). However, the use of *in vitro* vascular models with an endothelial lining is a feasible approach to study the association between biological changes and flow dynamics (Kaneko et al., 2018; Levitt et al., 2019; Jang et al., 2020).

The EC layer is a direct barrier that isolates blood flow from the vascular wall. It is sensitive to fluid shear stress and changes in shear force caused by local flow changes. In the physiological environment, arterial WSS is around 1–2 Pa, while venous WSS is around 0.1–0.6 Pa (Kamiya et al., 1984). When the WSS exceeds the physiological threshold, ECs undergo morphological changes. Therefore, the EC layer is the earliest receptor and effector of vascular remodeling. In addition, WSS-driven EC inflammation is the first step in aneurysm formation, and nuclear factor (NF)-*κ*B is a major inflammatory factor that mediates this step (Signorelli et al., 2018). Subsequently, proteolytic destruction of the vascular extracellular matrix by matrix metalloproteinases (MMPs) leads to aneurysm formation or rupture. MMPs and NF-*κ*B produced in the ECs are overexpressed in aneurysm tissue (Kataoka, 2015), which is evidence of inflammation in the aneurysm occurrence.

To investigate the correlation between flow shear variations induced by vascular stenosis and the morphology and function of ECs, we carried out the *in vitro* experiments of silicone stenotic artery populated by ECs and established the relation between the artery stenosis degree and the MMPs and NF-κB expression, and the relation between the MMPs and NF-κB expression and high/low WSS.

## Materials and Methods

### Fabrication of a Silicone Vascular Model

Straight transparent silicone vascular replicas with four degrees of stenosis (0, 30, 50, and 70%) were fabricated based on our brush-spin-coating method (Chi et al., 2021). An inner diameter of 5.5 mm was chosen based on the average diameter of the internal carotid artery (Liang et al., 2016). Vessel stenosis (*st*) was defined as follows: *st* = (1 − *d*/*D*) × 100%, where *d* and *D* are the diameters of the narrowest and normal regions, respectively.

**FIGURE 1:**
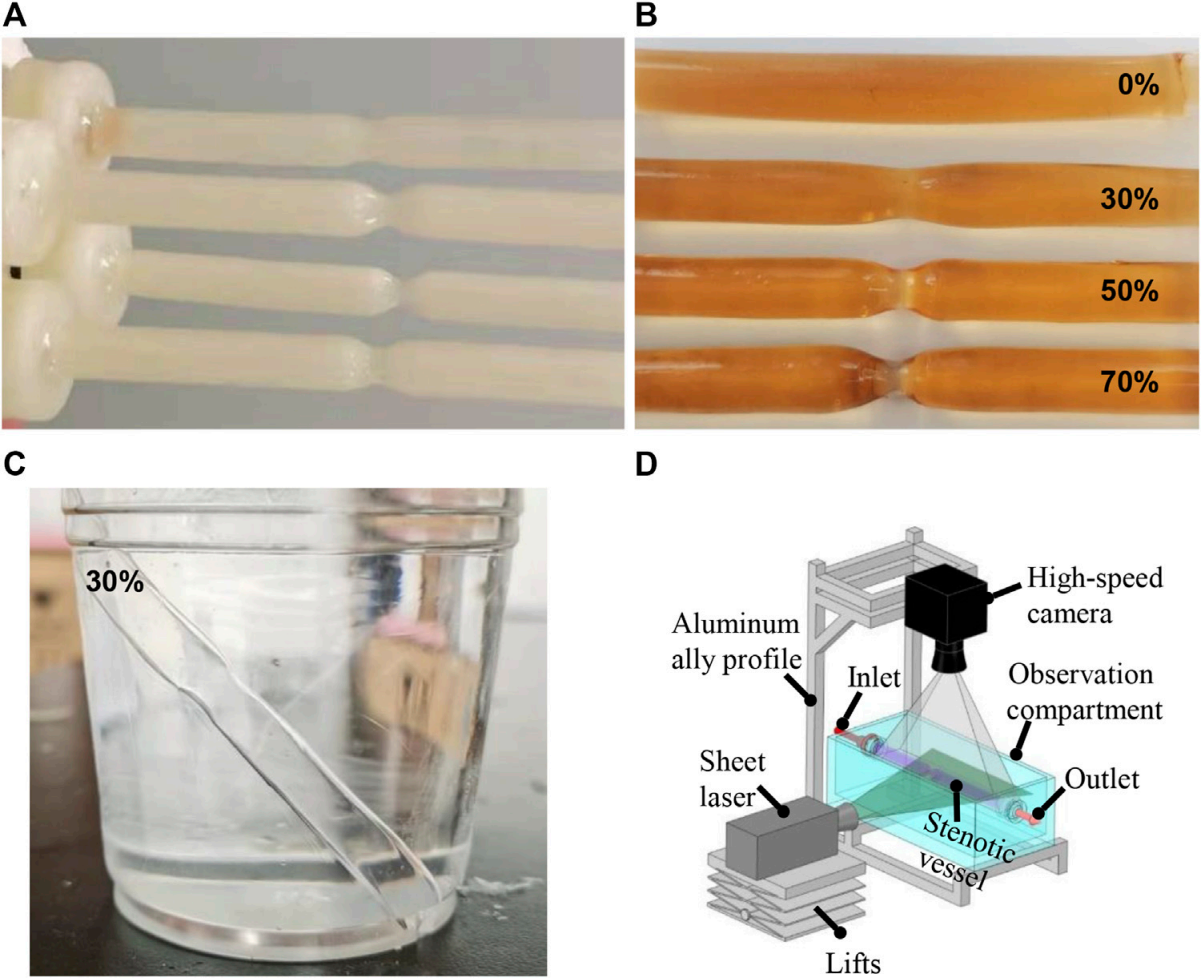
Water-soluble inner core **(A)** and silicone vessels **(B)** with different degrees of stenosis. Silicone tube in glycerol and aqueous solution with the weight ratio of 1:1 **(C)**. PIV experimental setup **(D)**.

Briefly, the vascular cores were first designed in ANSYS SpaceClaim (v17.0, ANSYS Inc., US) and saved as stereolithographical files, which were then imported into the bundled slicing software of a three-dimensional (3D) printer (Creator Pro, Zhejiang Flashforge 3D Technology, Zhejiang, China). The vascular cores of water-soluble poly(vinyl alcohol) (PVA) were generated (Figure 1A). The stair-like surfaces of these vascular cores were smoothened and coated with a transparent polydimethylsiloxane (PDMS; Sylgrad 184 Silicone Elastomer, US) solution to form a thin silicone layer, with a thickness of approximately 0.3 mm. A transparent silicone vascular replica was finally obtained by dissolving the PVA core in a water bath (Figure 1B). Figure 1C shows a silicone tube of 30% stenosis in glycerol and aqueous solution with a weight ratio of 1:1. When the silicone tube was submerged in the glycerol and aqueous solution, it shows a good transparency.

With consideration of that, the roughness of inner surface of the silicone tube is the main factor that influences the transparency of the silicone tube. In addition, it also makes a certain impact on the culture of ECs. Two operation specifications were adopted to smooth the inner surface of silicone tube and ensure the transparency of the silicone tube. The first step is to eliminate the stair-like texture of the water-soluble inner core. The water-soluble core is completely dipped into water 3 times, each time for 30 s, and the interval of 5 min after each dipping. The water will dissolve the texture on the surface of the core. After rinsing, the core is dried in a constant temperature oven at 40°C. And the second step is to further polish the inner surface of the silicone tube. The silicone tube was dipped into the same silicone liquid for 3 s, and a 0.7 MPa high-pressure air flow was then used to blow off the excess silicone on the wall of the tube for about 1 min, and then the treated silicone tube was dried at 60°C.

### Particle Image Velocimetry Analysis

As Varghese et al. (Varghese et al., 2007) reported that the flow field of the straight tube with a stenosis is extremely sensitive to the stenosis symmetry, and even a slight asymmetry can have a significant impact on the recirculation after stenosis and WSS distribution, which will differ from the CFD simulations in an extremely symmetric model. It will directly result in the variations of high-and low-WSS regions. Two smoothing treatments of silicone tube (as mentioned in *Section Fabrication of a Silicone Vascular Model*) may result in the asymmetry of the stenosis model to some extent. Hence, the main objective of the PIV study is to screen the stenosis vascular model with a symmetrical flow field and verify the CFD results.

PIV was performed using a high-speed camera (Photron, Fastcam-Mini UX 50, 2000 frames per second) with a continuous-pulse laser (MGL, 10 W, 532 nm, Changchun Institute of Laser Electronics, Changchun, China) for all the silicone vascular models before populating with human umbilical vein ECs (HUVECs) (Figure 1D). A solution of fluorescent beads was prepared containing 3.49% polyamide particles with a diameter of 20 μm suspended in glycerine aqueous solution with a weight ratio of 4:6. The bead solution was perfused through the silicone model using a peristaltic pump (BL600H, Baoding Zhunze Precision Pump Manufacturing Co. Ltd.) with a volumetric flow rate of 240 ml/min. PIV was focused on the predetermined positions with the stenosed region and its downstream region, and Z-plane imaging was performed through the central axis of the vessel. The PIV images were imported and processed using open source software (PIVLab in MATLAB) to screen the silicone vascular models with an axisymmetric flow and to validate the CFD simulation results.

### Cell Culture Within 3D Rotation and Flow State

The clean, sterilized, and fibronectin-coated silicone models were populated with HUVECs under 3D rotation. Each model was exposed to a culture medium under flow for 24 h to set up a perfusion cell culture.

**FIGURE 2:**
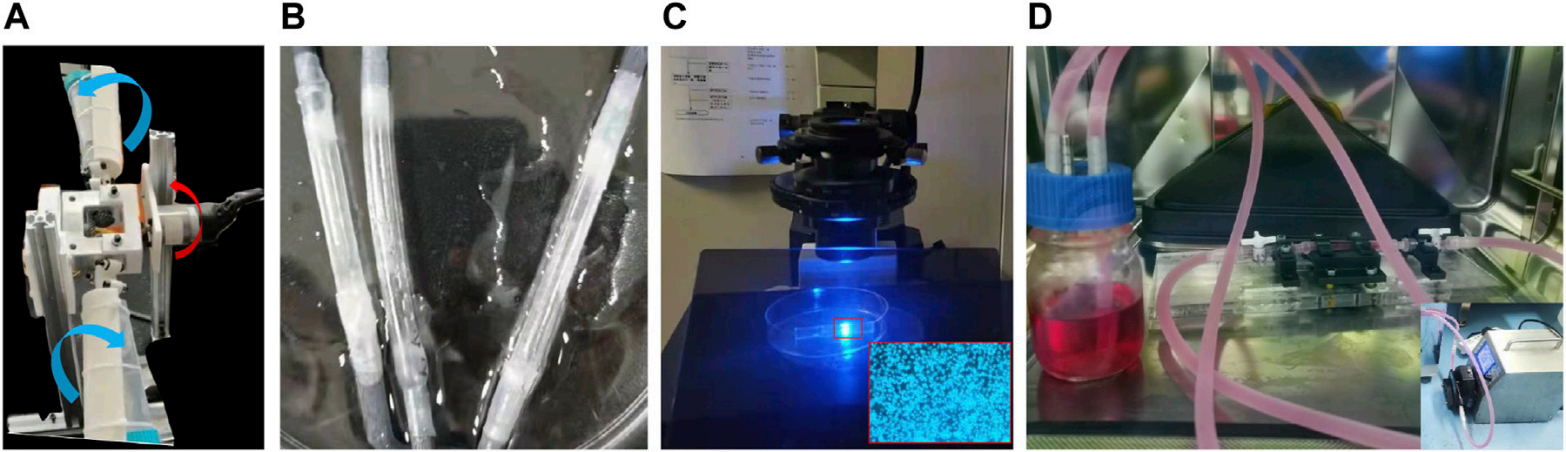
Biaxial rotating mechanism **(A)**, the endothelialized vascular model **(B)** and its observation under the inverted microscope **(C)**, and the device used for perfusion cell culture **(D)**.

When setting up the adherent cell culture, the surface of each silicone model was first cleaned with a 75% ethanol solution for 2 h followed by a plasma cleaner. The model was then sterilized on a sterile table by ultraviolet irradiation for 30 min. To enhance EC adhesion to the PDMS, the inner surfaces of the vascular replicas were twice coated with fibronectin at 40 μg/ml for 30 s and dried in a 37°C incubator. ECs were cultured in a growth medium comprising Eagle’s minimum essential medium, 10% fetal bovine serum, penicillin, and streptomycin. The silicone replicas were then immersed in a cell suspension (10^7^ cells/mL), packed into a centrifuge tube covered with a cap containing a filter for gas exchange, and rotated in a carbon dioxide (CO_2_) incubator at a rate of 0.25 rad/min with a biaxial rotating mechanism (Figure 2A). The two rotation directions are shown in blue and red arrows in Figure 2A. The HUVECs were adhered to the inner wall of the silicone vessels around 48 h later (Figure 2B), as shown in the inverted microscope (Olympus, IX73) image (Figure 2C).

During the perfusion cell culture, the endothelialized vascular models with different degrees of stenosis were connected to an experimental system consisting of silicone pipes, a peristaltic pump, and a liquid storage bottle with a gas exchange membrane. The EC culture medium, which contained 5% dextran (at a viscosity of 3.9 cPa), was delivered to the experimental system in a CO_2_ incubator (Figure 2D). An approximate steady flow rate of 240 ml/min (Tanaka et al., 2006) was provided by the peristaltic pump connected with a buffer for 24 h, and the experimental conditions were maintained for each degree of stenosis tested.

### Computational Fluid Dynamics Simulation

The geometric model of the stenosed artery used for the CFD simulation exactly replicated the model used for the experiment. Based on grid independence verification, a 0.2 mm mesh size was selected. The narrow part of the model required local mesh refinement, and the total number of grid points was approximately 700,000. The convergence standard of the Navier–Stokes equation was set to 10^–6^. The inlet velocity was derived from the experimental velocity, and a uniform inlet velocity boundary condition is applied. In order to obtain a fully developed inlet flow, the length of inlet section is taken as 35 mm in the geometric model, which is about 6.4 times the inner diameter of the tube. Blood was assumed to be an incompressible Newtonian fluid with a dynamic viscosity of 0.0035 Pa·s and a density of 1,060 kg/m^3^, and the wall was assumed to be rigid with no-slip boundary conditions. The outlet boundary was a zero-pressure outlet. The WSS and streamline distribution were then obtained.

**FIGURE 3:**
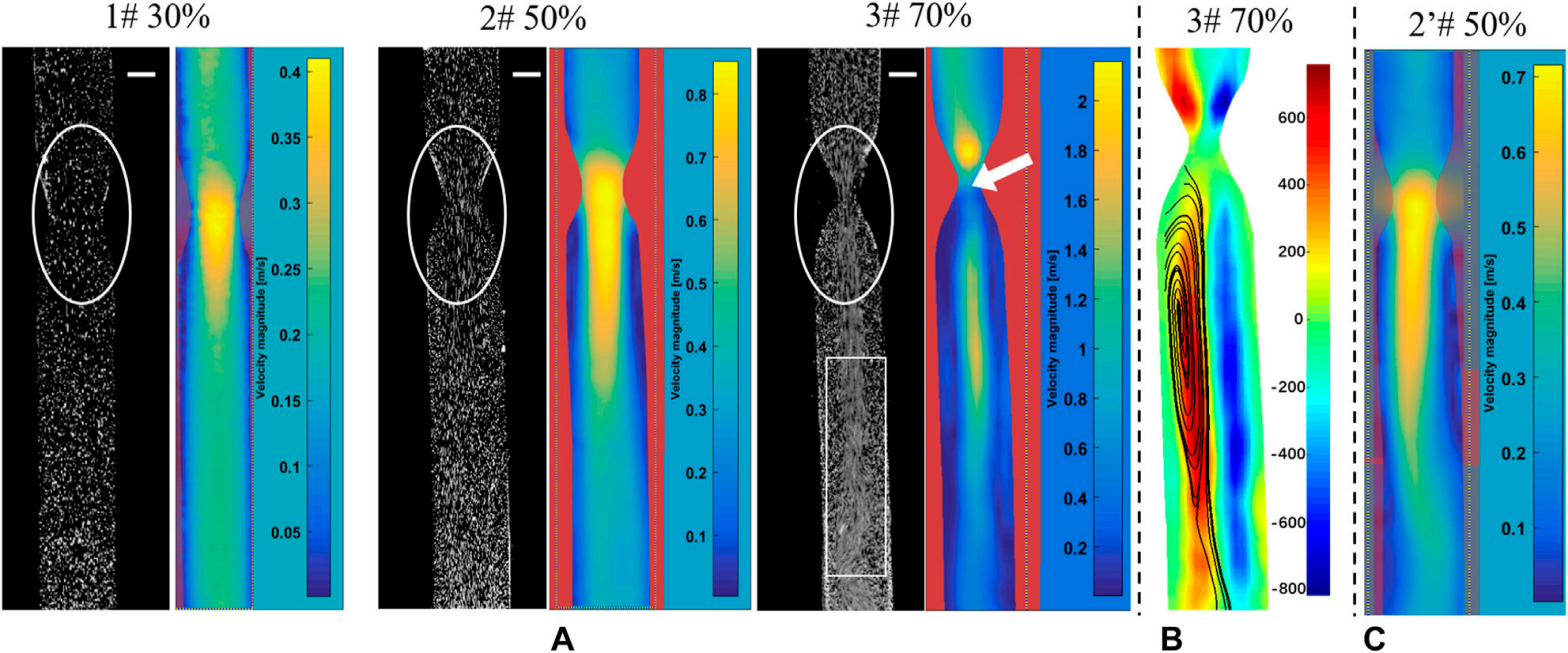
PIV measurements to screen for silicone vessel structures with good symmetry. The snapshots in PIV experiments and flow distribution **(A)**, vorticity distribution **(B)**, and flow distribution **(C)** in a vessel with 50% stenosis. The bar scale is 2 mm.

When the Reynolds number (Re) was <2,300, the blood flow state of the vessels was considered to be laminar, and the laminar flow model was set. However, the jet flow and flow separation occur in the downstream regions of stenosis, and severe stenosis can induce local turbulence (Varghese et al., 2007). For the 30 and 50% stenosis models, the laminar flow model was carried out. While for the 70% stenosis model, there was an evident jet flow in the narrow part (see the white circle, Figure 3A), and turbulent disturbance appeared due to the jet flow with much high flow rate, as the white box indicated in Figure 3A. With the consideration of the low Reynolds number, the SST *k*–*ω* model can simulate the simultaneous existence of laminar, transition, and turbulent flows more accurately than other laminar or turbulent models (Chi et al., 2021), the SST *k*-ω model was then adopted.

Therefore, the SST *k*-ω model was selected for the 70% stenosis model with a local Re number of >2,300 in ANSYS 2019 Fluent. This model can more accurately simulate the concomitant existence of laminar flow, transition flow, and turbulence (Wilcox, 1994).

### Endothelial Cell Morphometry and Gene Expression Analyses

ECs cultured on the inner surfaces of the vascular replicas were first washed with phosphate-buffered saline three times. Cultured ECs were then fixed in 4% paraformaldehyde. During immunofluorescent staining, the intracellular catalase was first removed using 3% hydrogen peroxide. Non-specific binding was blocked by incubating the cells with 1% rabbit serum (cat. ab7356; Abcam). The cells were stained with 4′,6-diamidino-2-phenylindole. The cells were then incubated with anti-mouse CD31 antibody (cat. 3528; CST) and either anti-rabbit NF-*κ*B antibody (cat. ab32536; Abcam) or anti-rabbit MMP-9 antibody (cat. ab76003; Abcam) in a moist box overnight at 4°C. The following day, the cells were treated with CoraLite488-conjugated goat anti-rabbit IgG antibody (cat. SA00013-2; Proteintech) and tetramethylrhodamine-conjugated goat anti-mouse IgG antibody (cat. SA0007-1; Proteintech). Each vascular replica was then cut into pieces to examine the different regions. Finally, the images of the samples were captured at 40 × magnification using a laser scanning confocal microscope.

## Results

### Particle Image Velocimetry Measurement

Figure 3A presents the snapshots in PIV experiments and the PIV post-processed results of flow distribution, which were used to examine the silicone vessel structure with good symmetry to exclude the asymmetric model caused by model smooth treatments. The stenosed silicone tubes (1#, 2#, and 3#) had a good symmetry in terms of the velocity distribution based on the post-processed PIV results. Figure 3B presents the vorticity distribution in the silicone vessel with 70% stenosis. The blue and red contours represent two vortices with reverse rotation with an almost symmetric distribution. Figure 3C shows the flow injection through the narrow part impinged on the sidewall of the tube, which was caused by asymmetric stenosis. It was reported that asymmetric stenosis affects the extent of post-stenotic recirculation and WSS distribution, which are sensitive to blood vessel geometry (Varghese et al., 2007; Haley et al., 2021). Hence, PIV measurements play an important role in screening the vascular model of stenosis with a symmetrical flow field.

In addition, the comparisons of experimental snapshots indicated in Figure 3A, which are with particles in different stenotic tubes captured using a high-speed camera. It can be seen that there is more evident jet flow in the narrow part with the stenosis degree increase, in particular in the model of 70% stenosis, as the white circle indicated. However, due to the limited frame rate of 2000 fps of high-speed camera in this experiment, there is a clear trailing effect of the moving particles, especially in the model of 70% stenosis. The particles are not easy to be captured in the high jet flow state, and the positions of particles are hard to be determined. It will be difficult for the PIV analysis based on the comparison of the relative position changes of particles at two adjacent moments. That is the reason why the high velocity distribution was not detected in the PIV result in the model with 70% stenosis, as the white arrow indicated.

### Validation of the Numerical Simulation Results

**FIGURE 4:**
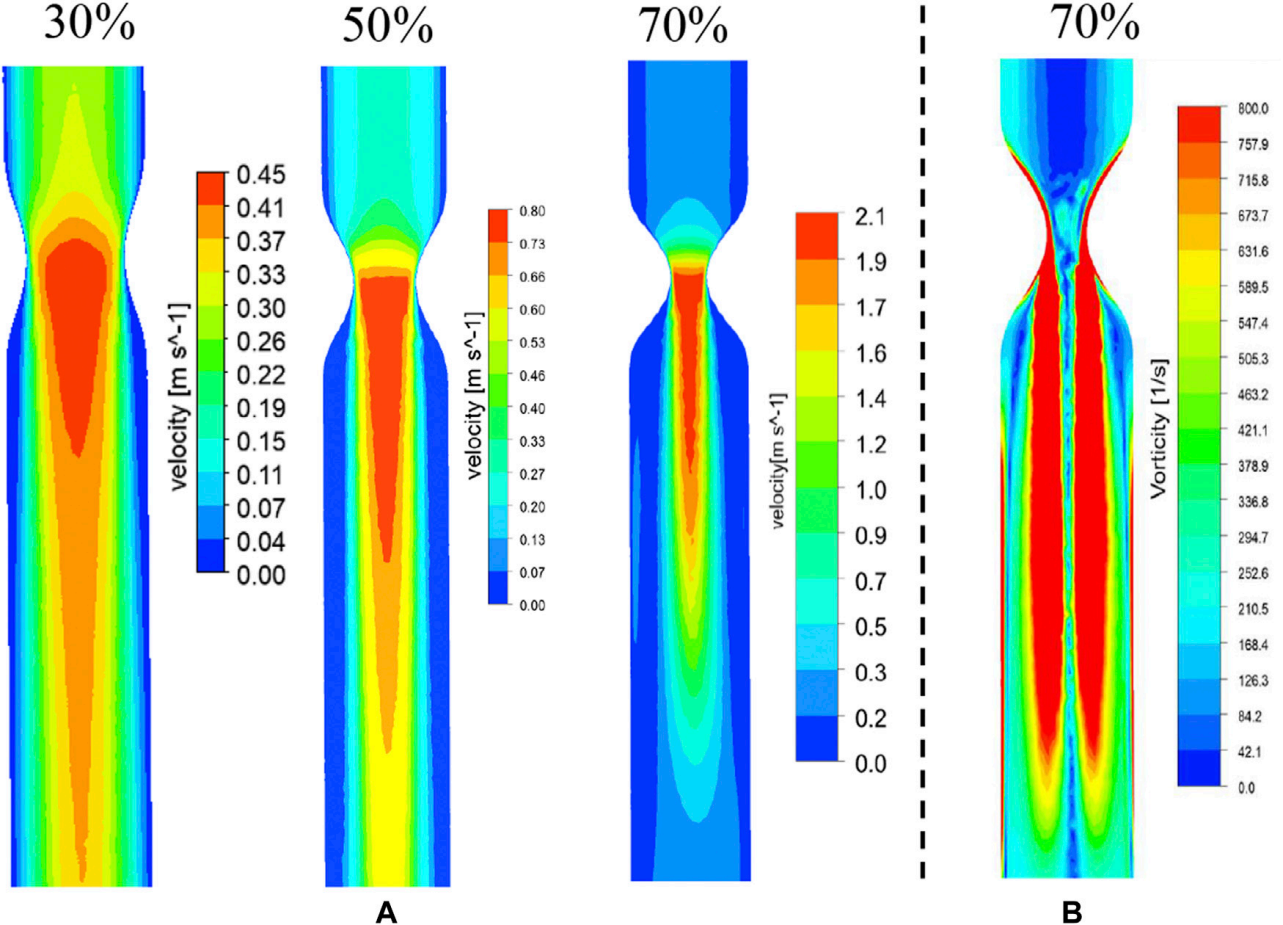
CFD results of flow **(A)** and vorticity **(B)** distribution in vascular structures with different degrees of stenosis.

Figure 4 shows the numerical simulation results of flow distribution in three vascular models of stenosis. The comparisons of flow and vorticity between the PIV (Figures 3A,B) and CFD (Figures 4A,B) results are also shown. The CFD results demonstrate good agreement with the PIV measurements in terms of the flow and vorticity distribution. Thus, the selection of the related fluid models for the numerical calculation is reasonable and reliable.

### Computational Fluid Dynamics Results

**FIGURE 5:**
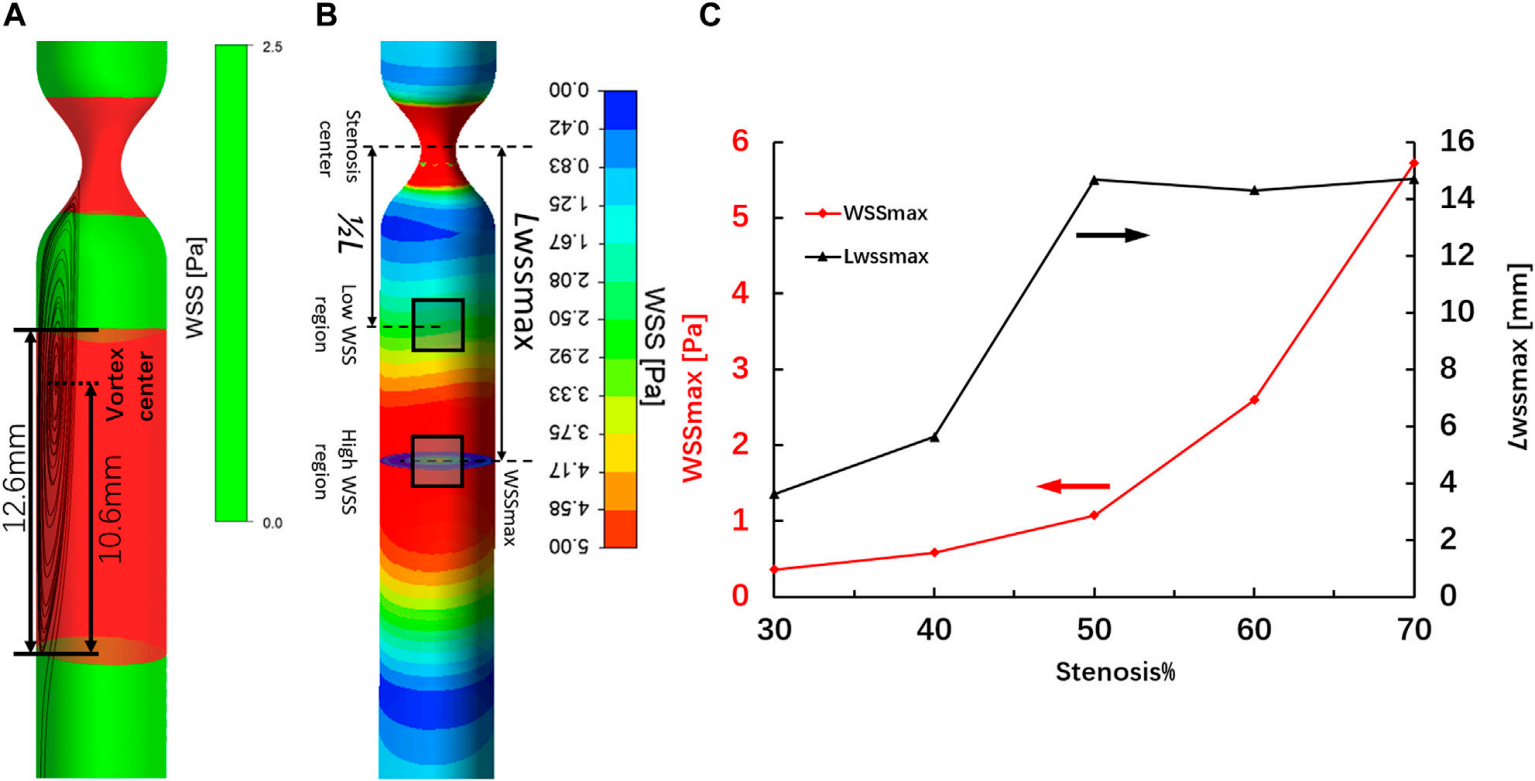
High-WSS region **(A)** and location of maximum WSS **(B)** in the region downstream of the artery with 70% stenosis. Location of maximum WSS and the value distribution in different stenosed structures **(C)**.

The WSS distribution and streamlines of the straight arteries with different degrees of stenosis were simulated by CFD using the same structural data used for 3D printing. Figure 5A shows the streamline and the WSS distribution in the downstream region of the vessel with 70% stenosis. The red contour shows the region in which the WSS values were >2.5 Pa. PIV revealed a clear vortex located downstream of the stenosed region (Figure 3B), and the high WSS was located close to the vortex. The distribution of WSS and the location of maximum WSS downstream of the 70% stenosed region are shown in Figure 5B. Figure 5C shows the location with the maximum WSS and the value distribution in different stenosed structures. The maximum WSS increased as the degree of stenosis increased. When the degree of stenosis was <50%, the maximum WSS was within 1.0 Pa. As the degree of stenosis reached 50%, the maximum WSS was >1.0 Pa. This value increased to >5.0 Pa when the degree of stenosis reached 70%. In addition, the location of maximum WSS first moved forward along the flow direction as the degree of stenosis increased, and backward against the flow after the degree of stenosis reached 50–70%. The maximum WSS was located 14–16 mm away from the center of the stenosed region.

### Endothelial Cell Morphology After the Flow Stimulation

**FIGURE 6:**
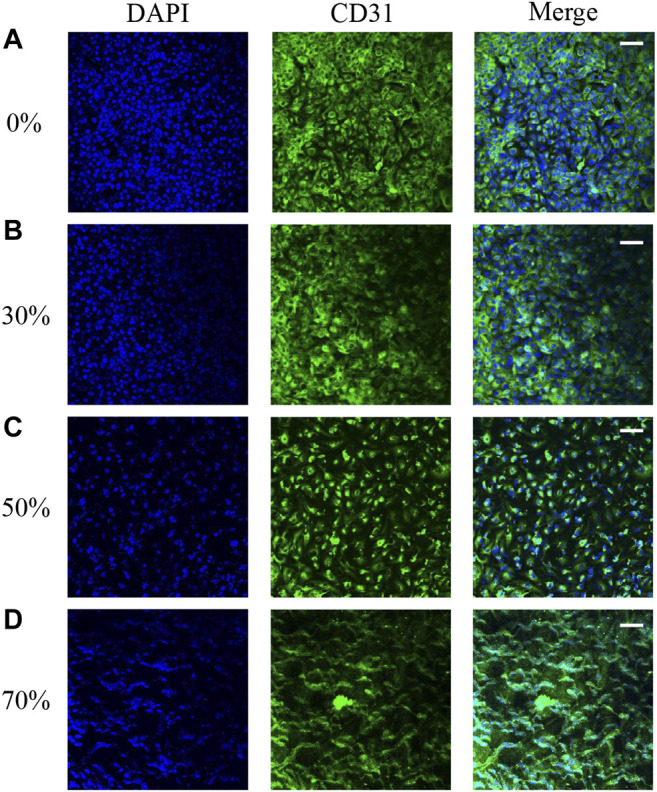
EC morphology in 0 **(A)**, 30 **(B)**, 50 **(C)**, and 70% **(D)** stenosed structures under flow stimulation.

Our hemodynamic analysis showed contrasting results in vortex regions with different degrees of stenosis. The changes in EC morphology after flow culture, especially in ECs located at the regions of maximum WSS downstream of stenosis in the vascular replica, were investigated by confocal microscopy. The microscopy images show that the cytoskeletal F-actin in ECs was stained with rhodamine–phalloidin after flow stimulation induced by different degrees of stenosis. The images also show that the inner surfaces of the silicone vessels with 0 and 30% stenosis were evenly covered with ECs (Figures 6A,B). As the degree of stenosis increased, the ECs located downstream of 50% stenosis became elongated in the longitudinal direction. The ECs located at regions of maximum WSS downstream of stenosis showed irregular alignment due to the existence of the vortex (Figures 6C,D). Moreover, the gaps between the cells increased as the degree of stenosis increased (Figures 6B–D). In particular, when the degree of vascular stenosis reached 70%, there were clear gaps between the cells, and the EC morphology clearly changed into an undulating-ribbon structure. Kamiya reported that in physiological environments, arterial WSS is around 1–2 Pa (Kamiya et al., 1984). The flow shear induced by stenosis of <50% does not exceed the physiological range that the cell is subjected to; thus, EC morphology does not change noticeably.

### Endothelial Cell Gene Expression After Flow Stimulation

**FIGURE 7:**
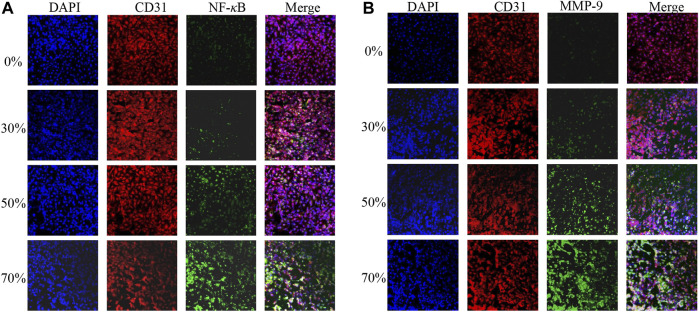
MMP-9 **(A)** and NF-κB **(B)** expression after different degrees of flow stimulation induced by stenosis.

We harvested ECs from the model of stenosis for gene expression analysis. The ECs were obtained downstream from the surrounding regions of maximum WSS after flow stimulation. The expression of two key vascular inflammatory factors (MMP-9 and NF-*κ*B) is shown in Figure 7. The expression of MMP-9 and NF-*κ*B in ECs from vessels with 0% stenosis did not differ from that in ECs from vessels with 30% stenosis. However, the expression of MMP-9 and NF-*κ*B appeared to be higher in regions downstream of higher WSS due to the greater degree of stenosis. In particular, in vessels with 70% stenosis, there was a significant increase in MMP-9 and NF-*κ*B expression. The increase in MMP-9 and NF-*κ*B expression was correlated with substantially high vortex regions and higher WSS values downstream of stenosis (Figure 5). A previous study showed that the expression of inflammatory factors is consistent with variations in EC morphology, which can be explained by a stenosis degree of >50%, and WSS induced by stenosis exceeds the physiological range of the cell (Kamiya et al., 1984).

**FIGURE 8:**
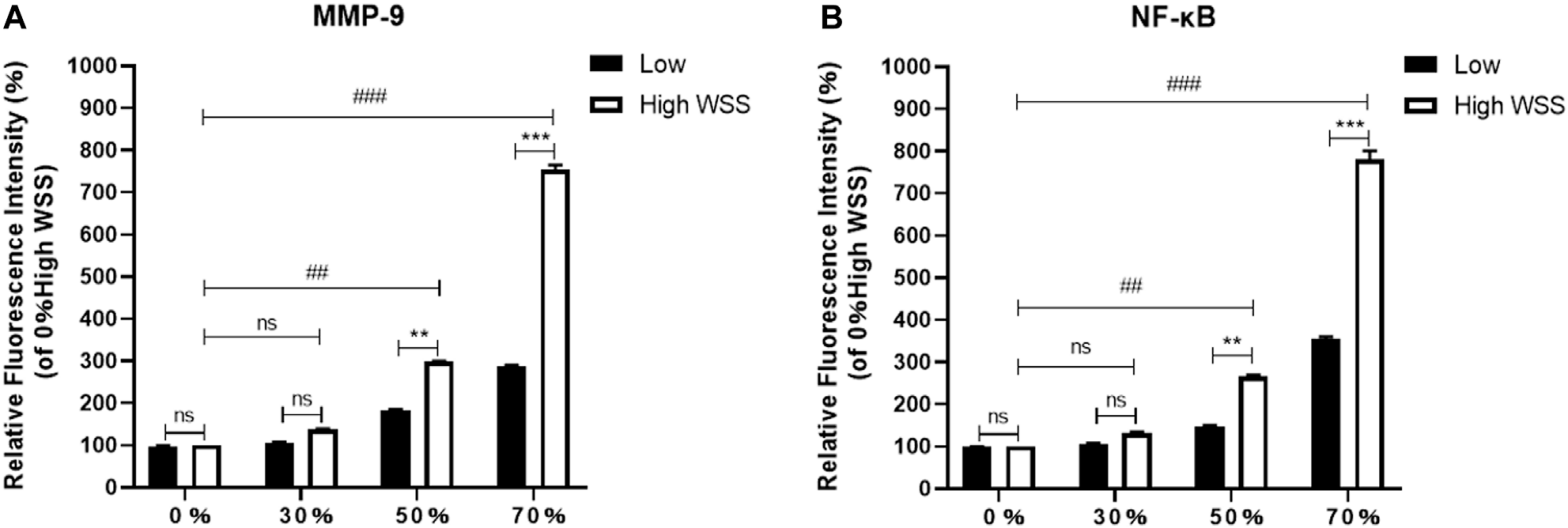
Comparison of relative fluorescence intensity of MMP-9 **(A)** and NF-κB **(B)** at the high- and low-WSS regions in different stenosis models.

In addition, we further compared the relative fluorescence intensities of MMP-9 and NF-*κ*B at the high-WSS (adjacent to the maximum WSS location, ∼ *L*_WSSmax_) and low-WSS regions (∼1/2 *L*_WSSmax_) as indicated in Figure 5B. The relative fluorescence intensities of MMP-9 and NF-*κ*B were obtained by comparing the fluorescence intensity of current images with the one in the model of 0% stenosis located high-WSS region. As Figures 8A,B indicated, the expression levels of MMP-9 and NF-*κ*B show an increased tendency with the increase in stenosis degree, and for the model of 0 and 30% stenosis, there is no clear difference between the high- and low-WSS regions. However, at 50 and 70% stenotic model, the expression levels of MMP-9 and NF-*κ*B located at the high- and low-WSS regions show significant variations. It means that the changes in flow shear stimulation induced by stenotic arteries, in particular high WSS, will directly affect the expression of inflammatory factors which will participate in the process of vascular dilation. The fluorescence intensity of the image is calculated using the software of ImageJ (ImageJ 1.4.3.67, http://imagej.nih.gov/ij), and the data was obtained by GraphPad software (GraphPad Prism 8.0.1, https://www.graphpad-prism.cn).

## Discussion

In this study, we successfully created a monolayer of ECs in a silicone tube, as described in previous studies (Kaneko et al., 2018; Levitt et al., 2019). To ensure that the inner surfaces of the silicone vessel models were evenly covered with ECs and to easily obtain focused confocal images, the roughness and thickness of the silicone tubes, the EC concentration, and the speed of rotation were standardized.

Although fabrication of silicone vessel replicas has been described previously (Chi et al., 2021), the standardization of inner tube roughness and silicone tube thickness in this study are noteworthy. A stair-like texture of the water-soluble skeleton is difficult to avoid due to the layer-by-layer printing technique. This is not desirable as ECs are cultured and regionalized based on the surface texture. Thus, the water-soluble skeleton surface needs to be fully smoothened. However, as reported by Heley et al., the symmetric post-stenotic recirculation and WSS distribution depend on the symmetry of the stenosed vessel. The smoothing process will affect the extent of silicone vessel stenosis. Thus, it is important to screen the symmetry of vascular replicas based on PIV measurements. The thickness of the silicone replica was maintained at <0.5 mm to ensure higher transparency and better tiling of the thin silicone pieces to improve image quality by confocal microscopy. In addition, it is important to determine the appropriate cell suspension density and rotation speed to obtain evenly spread ECs. High-density suspensions and low rotation speeds can reduce the number of cells stacked together, whereas low-density suspensions and high rotation speeds can prevent ECs from adhering to the silicone wall.

Based on the CFD simulation, the WSS/WSSG and the location of maximum WSS were easily identified. The maximum WSS increased with the degree of stenosis. In addition, the location of maximum WSS fluctuated between 12 and 16 mm from the site of stenosis and was farthest at 50% stenosis. This was dependent on changes in the location of the vortex center. Our results are in good agreement with previous statistical measurements of the distance between the narrow region and the aneurysm neck (Antonov et al., 2018).

We found that substantial changes in EC morphology and high expression of major inflammatory factors were directly related to high WSS, which may be a key factor mediating aneurysm occurrence. Our results are in line with previous observations showing that aneurysms predominantly occur at regions of high WSS (Geers et al., 2017). Excessive and abnormal WSS can also cause dysfunction or loss of the endodermis, which can lead to local vascular wall degeneration and expansion (Meng et al., 2014; Kataoka, 2015; Staarmann et al., 2019). In addition, it is known that the MMPs and NF-κB inflammation cause artery dilation. In the current study, *in vitro* experiments of silicone stenotic artery with different stenosis degrees populated by ECs were carried out, a direct relation between the artery stenosis degree and the MMPs and NF-κB expression was clearly established. The expression levels of MMP-9 and NF-κB located at the high-WSS region show significant variations in the 50 and 70% stenotic models. In addition, the expression levels of MMP-9 and NF-κB located at the high- and low-WSS regions show a significant variation in a 50% or 70% stenotic model. It means that the stenotic artery over 50% stenosis could be a potential risk factor for the post-stenotic dilation, which would pay more attention to clinical diagnosis.

This study has several limitations that should be noted. First, the physiological composition of the blood vessel wall is much more complex than that of the simplified silicone and EC layers used in our model. Second, the boundary condition setting might influence the simulation results. A steady average flow rate over a period of time in the carotid artery was set as the inlet flow condition without considering the real pulsatile wave of the blood. The differences between the steady and pulsatile inflow conditions would have influenced our WSS calculations in the CFD simulation. We set a zero-pressure outlet as the outlet boundary. If the experimentally measured pressure was adopted as the outlet boundary, the CFD result will surely make a better matching degree of experimental result in this model. Third, the frame rate of the high-speed camera is set to 2000 fps. Within the limited frame rate, there is a clear trailing effect of the moving particles. It will bring trouble in the PIV analysis based on the comparison of the relative position changes of particles at two adjacent moments. It might be difficult to capture the jet flow in PIV post-processed software of PIVLab.

## Conclusion

*In vitro* experiments and CFD simulations revealed that hemodynamic stress variations induced by arterial stenosis contribute to differences in EC morphology and gene expression. A high degree of vascular stenosis can directly give rise to high WSS downstream of the stenosed region, leading to the appearance of gaps between ECs and an increase in the expression of key inflammatory factors. These inflammatory factors, which are produced by ECs, are also commonly found in aneurysm tissue. This study will be helpful for our understanding of the mechanobiological mechanism underlying the link between vascular stenosis and post-stenotic dilation.

## Data Availability

The original contributions presented in the study are included in the article/Supplementary Material, further inquiries can be directed to the corresponding author.
